# Changing monoclonal antibody keeping unaltered the chemotherapy regimen in metastatic colorectal cancer patients: is efficacy maintained?

**DOI:** 10.1186/2193-1801-2-185

**Published:** 2013-04-25

**Authors:** Roberta Grande, Donatello Gemma, Isabella Sperduti, Alain Gelibter, Maria Anna Giampaolo, Giorgio Trombetta, Fabrizio Nelli, Teresa Gamucci

**Affiliations:** 1 Medical Oncology Unit, ASL Frosinone, Kragujevac, Italy; 2 Bio-Statistics Unit Regina Elena National Cancer Institute, Rome, Italy; 3 Medical Oncology Unit Regina Elena National Cancer Institute, Rome, Italy; 4 Medical Oncology Unit Belcolle Hospital, Viterbo, Italy

**Keywords:** Metastatic colorectal cancer, Bevacizumab, Cetuximab, Chemotherapy, Monoclonal antibody

## Abstract

Monoclonal antibodies bevacizumab and cetuximab both improve overall survival (OS), progression free survival (PFS) and overall response rate (ORR) when combined with irinotecan-containing regimens. The optimal sequence of these monoclonal antibodies in combination with chemotherapy is controversial. This study analysed the efficacy of cetuximab plus Folfiri after progression with the same regimen plus bevacizumab in patients with metastatic colorectal cancer (mCRC). Patients are eligible if progressive disease (PD) after Folfiri-bevacizumab; ECOG PS 0–1. Primary endpoint is the disease control rate (DCR:ORR plus stable disease > 6 months); secondary endpoints: ORR, PFS, duration of response, OS and toxicity. ORR and DCR were reported with their confidence interval at 95%. Kaplan-Meier method was used for PFS and OS evaluation. Results: 54 patients were enrolled to receive Folfiri-cetuximab after PD to Folfiri-bevacizumab treatment. Median age was 65 (43–80), M/F 31/23, ECOG PS 0/1 was 36/ 18, WT Kras 33(61%). The DCR was 64.8% (CI 95% 52.1-77.5). Among the group of patients with stable or progressive disease at first line treatment, 13.3% of them obtained a response at second line. For second line treatment median duration of response was 6 months and clinical benefit 7 months. The ORR was 22.2% (CI 95% 11.1-33.3). The median progression-free survival was 7 months (CI 95% 6–8). The median overall survival for second line treatment was 14 months (CI 95% 11–17). No grade 4 toxicity was observed. Data suggest that this sequential combination therapy is active and well tolerated. At disease progression to first line chemotherapy treatment the maintenance of the same chemotherapy regimen and the change of the monoclonal antibody showed efficacy in response and survival in patients with mCRC.

## Introduction

Colorectal cancer is a major cause of morbidity and mortality worldwide. Nowadays the median duration of survival among patients with advanced colorectal cancer has been increased through the introduction of new drugs. Survival has reached about twenty-four months (Van Cutsem et al. 2009).

These significant improvements are the result of new combinations of standard drugs, such as fluorouracil, irinotecan and oxaliplatin, in association with new therapeutic agents, namely bevacizumab and cetuximab (De Gramont et al. 2000; Douillard et al. 2000a; Giacchetti et al. 2000; Goldberg et al. 2004a; Saltz et al. 2000a; Cunningham et al. 1998; Rougier et al. 1998; Cunningham et al. 2004a).

These monoclonal antibodies target molecular regions involved in colorectal carcinogenesis. Bevacizumab, a humanized monoclonal antibody directed against vascular endothelial growth factor A (VEGF-A), improves response rate (RR), progression-free survival (PFS) and overall survival (OS) in combination with irinotecan when compared with patients treated with chemotherapy treatment alone (Hurwitz et al. 2004). Similar benefits in OS, PFS and RR were also shown in second line treatment , where adding bevacizumab to standard chemotherapy (Giantonio et al. 2007).

Moreover, cetuximab, a human-murine chimeric monoclonal antibody that targets epidermal growth factor receptors (EGFR), has shown antitumor activity both alone and in combination with irinotecan (Cunningham et al. 2004b; Saltz et al. 2001; Saltz et al. 2004; Lenz et al. 2006).

Until last year standard first-line chemotherapy included fluorouracil with leucovorin and irinotecan or oxaliplatin, alone or combined with bevacizumab (Douillard et al. 2000b; Saltz et al. 2000b; Goldberg et al. 2004b ; Van Cutsem & Geboes 2007).

Recently, CRYSTAL and OPUS studies have shown the activity of cetuximab in first line chemotherapy treatment in wild-type KRAS gene status. This gene is predictor for resistance to epidermal growth factor receptor (EGFR) monoclonal antibody therapies (Van Cutsem et al. 2009; Bokemeyer et al. 2009; McLellan et al. 1993; Arber et al. 2000; De Roock et al. 2008; Di Fiore et al. 2007b; Lièvre et al. 2008; Lièvre et al. 2006; Cervantes et al. 2008).

However, the optimal sequence of these monoclonal antibodies in combination with chemotherapy is controversial and there are no studies suggesting what the most effective sequence of these drugs is.

Therefore this study aims to evaluate the feasibility of a sequential chemotherapy regimen. In particular it aims to explore the efficacy of cetuximab in association with irinotecan-based chemotherapy (Folfiri) after disease progression with the same chemotherapy regimen plus bevacizumab in patients with metastatic colorectal cancer.

## Materials and methods

Patients were considered eligible if they had pathologically confirmed colorectal cancer, metastatic disease, positive EGFR immunostaining. To be eligible, patients must have received at least six months of first line chemotherapy with Folfiri plus bevacizumab. Eligibility criteria also included: age ≥18 years, Eastern Cooperative Oncology Group (ECOG) performance status of ≤1; normal hematopoietic function (haemoglobin, at least 9 g per decilitre [5.6 mmol per liter]; neutrophil count, at least 1500 per cubic millimeter; and platelet count, at least 100,000 per cubic millimeter), renal function (serum creatinine, less than 1.5 times the upper limit of normal), and liver function (bilirubin, not more than 1.5 times the upper limit of normal; aspartate aminotransferase and alanine aminotransferase, not more than 5 times the upper limit of normal); no coexisting medical problem of sufficient severity to limit study compliance.

### Dosage and drug administration

EGFR status of the tumor was determined by immunoistochemical analysis of a paraffin-embedded tumor specimen with the use of an EGFR diagnostic kit (Dako Cytomation). Since 2006 attention has been focused on intracellular mediators involved in the transduction of EGFR signal to predict the efficacy of the treatment and KRAS pathways have been investigated. (Lievre et al. 2006; De Roock et al. 2007; Di Fiore et al. 2007a; Khambata-Ford et al. 2007 ; Lievre et al. 2008). From November 2008 we had the opportunity to analyse KRAS oncogene status by the PCR/sequencing technique.

After screening, patients were enrolled to receive first line chemotherapy with Folfiri (irinotecan 180 mg/m^2^ IV over 90 minutes, day 1, concurrently with folinic acid 100 mg/m^2^ day 1–2 IV over 120 minutes followed by fluorouracil 400 mg/m^2^ IV bolus, days 1,2, then fluorouracil 1200 mg/m^2^/die intravenous infusion over 46 hours) plus bevacizumab 5 mg/Kg day1. Treatment was administered every 2 weeks. If patients maintained at least stable disease after 6 months of treatment they continued therapy with bevacizumab 7.5 mg/Kg every 3 weeks as maintenance.

Patients showing disease progression underwent to second line treatment with the same chemotherapy schedule, but replacing monoclonal antibody with cetuximab (first dose 400 mg/mq then 250 mg/mq weekly). If stable disease was maintained after 6 months, patients continued therapy with cetuximab alone with a dosage of 250 mg/mq weekly until intolerable toxicity or progressive disease.

Disease progression was documented by computed tomography (CT), magnetic resonance imaging (MRI) or Positron Emission Tomography (PET).

Toxic effects were assessed according to the National Cancer Institute Common Toxicity Criteria, version 3. Modifications of the dose were made in cases of hematologic or non-hematologic toxic effects.

The primary endpoint was the disease control rate (DCR) defined as ORR plus stable disease ≥6 months. The secondary endpoints included the overall response rate (ORR), the progression-free survival (PFS), the duration of response, the overall survival (OS) and the toxicities.

All patients signed a consent form. The study was approved by the institutional ethic committee.

### Statistical analysis

The endpoint of this study is the disease control rate (DRC defined as ORR plus stable disease ≥6 months).

This phase II trial is planned as a single-stage design as described by A’Hern. A sample of 53 patients is considered sufficient to give an 80% probability of rejecting a baseline disease control rate (DCR) of 35% with an exact 5% one-sided significance test when the true DCR is 55%. The drug regimen will be rejected if less than 25 DCR (as previously defined) are observed.

All patients enrolled were considered the intention-to-treat population (ITT). This population was evaluated for the efficacy and safety analysis.

The standard summary statistics were used for both continuous and discrete variables. The DCR and the objective response rate were reported with its 95% confidence interval.

The time to event analysis was performed according the Kaplan-Meier product-limit method. The log-rank test was used to assess differences between subgroups. Significance was defined at the p<0.05 level.

## Results

Between January 2008 to January 2010, 54 patients with positive EGFR status after progression of the disease at first line with Folfiri plus bevacizumab were enrolled to receive second line combination treatment with Folfiri plus cetuximab (FC) followed by cetuximab (C) alone if stable disease was maintained. The analysis of Kras status was feasible and performed for 33 (61%) tumor specimens. Patient’s characteristics are shown in Table 1.

**Table 1:** **Baseline patient’s characteristics**

Characteristics (N=54)	N° of patients (%)
Median age (range)	65 (43–80)
Median follow-up from 1^st^ line, months (range)	26 (8–65)
Median follow-up from 2^nd^ line, months (range)	13 (3–51)
Sex Men	31 (57)
Women	23 (43)
ECOG performance status	
0/1	36 (67)/18 (33)
Primary diagnosis	
Colon cancer	42 (78)
Rectal cancer	12 (22)
Metastasis Site	
Single	41 (76)
Multiple	13 (24)
KRAS status	
Wild type (%)	31 (57)
Mutant Kras (%)	2 (4)
Unknown	21 (39)

Median cycles of first line treatment were 12 (range 4–16) for 35 patients and 8 (1–23) cycles of bevacizumab alone for 32 patients with at least stable disease.

Median cycles of second line chemotherapy with Folfiri plus cetuximab were 9 (range 1–14) for 19 patients and 7 (range 2–15) for 12 patients that continued treatment with cetuximab alone for maintenance.

**Figure 1:**
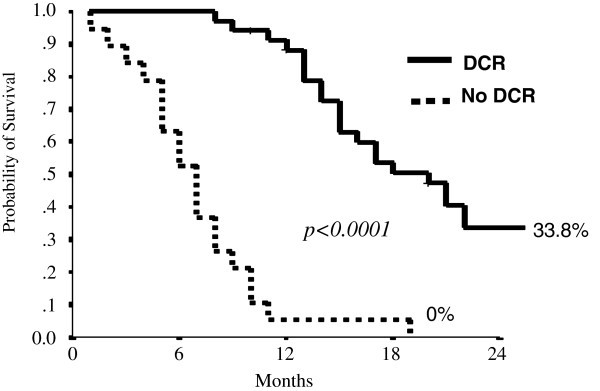
**DCR survival curve for second line treatment (Kaplan Maier Method).**

The Disease Control Rate was achieved in 64.8 percent (CI 95% 52.1-77.5) of the patients who received second line treatment with Folfiri plus cetuximab (Figure 1 and Table 2).

**Table 2:** **Kaplan Maier estimate for overall response rate (ORR) and disease control rate (DCR)**

	Overall survival				
	6 months	12 months	24 months	Median (CI 95%)	p value
ORR					0.02
No	78.6	49.7	11.8	12(9–15)	
Yes	100	91.7	55.0	27(11–43)	
DCR					
No	52.3	5.3	-	7(5–9)	<0.0001
Yes	100	88.2	33.8	20(15–24)	

The overall response rate (the rate of complete response plus the rate of partial response, ORR) was 22.2 percent (95% confidence interval, 11.1 to 33.3 percent) (Table 3).

**Table 3:** **Overall response rate**

	First line	Second line
	N. (%)	N. (%)
Best Overall Response	22 (44.5)	12 (22.2)*
Complete Response	5 (9.3)	0 (0)
Partial Response	19 (35.2)	12 (22.2)
Stable Disease	22(40.7)	23 (46.2)
Progressive Disease	8 (14.8)	19 (35.2)
Disease Control Rate	44 (81.5)	35 (64.8)**
* CI 95% 11.1-33.3		
**CI 95% 52.1-77.5		

At first line chemotherapy 44.5% of the patients had a complete or partial response; 33.3% of them continued to be responsive to second line with the same chemotherapy regimen plus cetuximab. Of special interest is the finding that 13.3% of patients with stable or progressive disease obtained a response.

For second line treatment the median duration of response was 6 months (CI 95% 4–8). The clinical benefit was 7 months (range 1–21).

**Figure 2:**
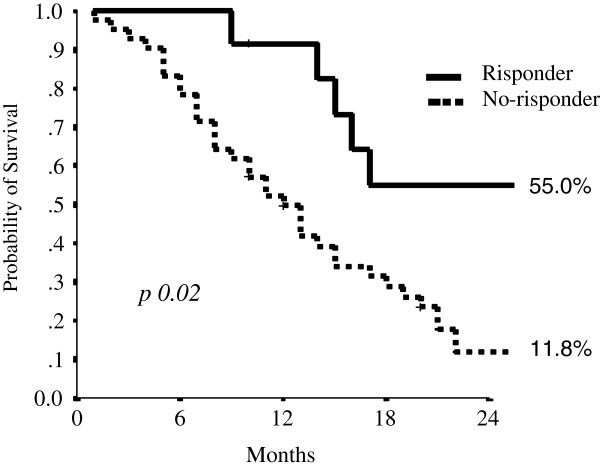
**Responder survival curve for second line therapy (Kaplan Maier Method).**

The median progression-free survival was 7 months (CI 95% 6–8). The percentage of patients free of progression at year one was 15.9%. The median overall survival for second line treatment was 14 months (CI 95% 11–17). At year one 58.9 percent of patients were alive and 21.8 percent at years two (Figure 2 and Table 2).

The median OS of all patients from first line chemotherapy with Folfiri plus bevacizumab was twenty-seven months (CI 95% 25–29). The overall survival for 32 (59.3%) patients that had DCR at first and second line treatment was 33 months (CI95% 26.4-39.6).

In Table 4 and 5 toxicities were shown. The majority of patients presented grade 1–2 side effects.

**Table 4:** **Main adverse events at first line chemotherapy**

	Grading				
	0	1	2	3	4
Neutropenia	25	11	6	9	3
Anaemia	41	13	0	0	0
Skin toxicity	0	1	0	0	0
Fatigue	29	10	13	2	0
Nausea/Vomiting	40	13	1	0	0
Hypertension	43	10	1	0	0
Bleeding	41	13	0	0	0
Proteinuria	49	3	2	0	0
Diarrhea	29	12	11	2	0
Stomatitis	37	6	11	0	0

**Table 5:** **Main adverse events at second line chemotherapy**

	Grading				
	0	1	2	3	4
Neutropenia	39	7	6	2	0
Anaemia	40	12	1	1	0
Skin toxicity	16	17	17	4	0
Fatigue	37	8	8	1	0
Nausea/vomiting	46	8	0	0	0
Hypertension	54	0	0	0	0
Bleeding	54	0	0	0	0
Proteinuria	54	0	0	0	0
Diarrhea	28	14	11	1	0
Stomatitis	34	10	12	0	0

Data showed that no increasing of toxicities was noted moving from first line to second line treatment although patients restarted the same chemotherapy regimen.

First line chemotherapy with Folfiri plus bevacizumab was well tolerated. Only 3 patients had grade 4 neutropenia. No other grade 4 toxicities were presented. No bleeding, thromboembolism or severe hypertension were shown. Dose reduction of irinotecan was made for only three patients for side effects reasons.

In the group of patients treated with cetuximab acne-like rash occurred in 70% of patients (39% grade ≥2; no grade 4 toxicity was observed). No severe gastrointestinal side effects were shown.

57 percent of patients reached and made it to third line chemotherapy. Due to acceptable performance status 15 patients were administered to FOLFOX4 chemotherapy regimen (standard dose or adjusted dose if necessary). For eight patients only monotherapy regimen with fluoropyrimidines was possible.

To date nine patients are alive and two of them are still in treatment with chemotherapy.

## Discussion

The analysis of literature shows that metastatic colorectal cancer patients, who have received chemotherapy may survive for about twenty-four months. If the effective drugs have failed, there are no other options (Cunningham et al. 2004a; Hurwitz et al. 2004).

This study analysed a feasible sequence of monoclonal antibodies maintaining the same chemotherapy regimen. The results confirmed the feasibility and efficacy of treatment.

In accordance with the outcome of phase III trials the response rate in first line treatment reached about 44% and second line showed about 22% (Cunningham et al. 2004a; Hurwitz et al. 2004).

The changing of bevacizumab after progression of disease with cetuximab produced an interesting survival rate in this sample of patients without resulting in an increase of toxicities.

Indeed, the median overall survival from first line treatment was 27 months, that reached 33 months for patients that maintained DCR at first and second line therapy.

From first line treatment 8 (14.8%) patients had progressive disease at six months of chemotherapy and started second line chemotherapy. Among them, 6 patients had progressive disease at Folfiri plus cetuximab and the resulting overall survival was 14 months for these patients. The other two patients had clinical benefit (DCR) at second line treatment of 14 and 15 months respectively and the overall survival was 21 and 23 months respectively. The clinical benefit was 7 months (range 1–21).

The achievement of a new responsiveness in these progressive patients had an impact on survival. This result was allowed by the changing monoclonal antibody.

Despite the use of the same chemotherapy for about twelve consecutive months, the regimen was well tolerated and there was no evidence of increase of the frequency or severity of the characteristic toxicities associated with treatment.

Moreover, it is very important to emphasize that the population included also older patients with 21.5% of them being older than 70. This characteristic did not influence the results concerning toxicities and survival rates.

In this study the number of patients receiving third line chemotherapy is encouraging (57 percent) and it underlines the feasibility of this sequence, which helps an acceptable quality of life for patients.

These data suggest that this sequential combination therapy is active, however, due to the small number of patients this study is limited and the KRAS mutation status was not available for all patients.

Furthermore, it should also be noted that at the beginning of the study a high percentage of patients (76%) had only a single distant metastatic site.

When the study started no data were available for the efficacy of cetuximab plus chemotherapy in the first line treatment of metastatic colorecatal cancer.

Recently, first line treatment with cetuximab has been approved in combination with chemotherapy and the benefits of cetuximab was limited to patients with KRAS wild-type tumors, reducing the risk of progression of metastatic colorectal cancer.

In this study for only 33 patients the analysis of KRAS status was performed; an adequate selection of patients could improve survival.

In conclusion, this research underlines the need for more information about a better sequence of chemotherapy regimens for this kind of patients.

It would be interesting to evaluate in a larger randomized trial what is the better sequence of drugs that can allow patients to obtain better responses and consequently a longer survival along with an acceptable quality of life.

## References

[CR1_242] Arber N (2000). Activation of c-K-ras mutations in human gastrointestinal tumors. Gastroenterology.

[CR2_242] Bokemeyer C (2009). Fluorouracil, leucovorin, and oxaliplatin with and without cetuximab in the first-line treatment of metastatic colorectal cancer. J Clin Oncol.

[CR3_242] Cervantes A (2008). Correlation of KRAS status (wild type [wt] vs. mutant [mt]) with efficacy to first-line cetuximab in a study of cetuximab single agent followed by cetuximab + FOLFIRI in patients (pts) with metastatic colorectal cancer (mCRC). J Clin Oncol.

[CR4_242] Cunningham D (1998). Randomised trial of irinotecan plus supportive care versus supportive care alone after fluorouracil failure for patients with metastatic colorectal cancer. Lancet.

[CR5_242] Cunningham D (2004). Cetuximab monotherapy and cetuximab plus irinotecan in irinotecan-refractory metastatic colorectal cancer. N Engl J Med.

[CR6_242] De Gramont A (2000). Leucovorin and fluorouracil with or without oxaliplatin as first-line treatment in advanced colorectal cancer. J Clin Oncol.

[CR7_242] De Roock W (2007). *Kras* wild-type state predicts survival and is associated to early radiological response in metastatic colorectal cancer treated with cetuximab. Ann Oncol.

[CR8_242] De Roock W (2008). KRAS wild-type state predicts survival and is associated to early radiological response in metastatic colorectal cancer treated with cetuximab. Ann Oncol.

[CR9_242] Di Fiore F (2007). Clinical relevance of *kras* mutation detection in metastatic colorectal cancer treated by cetuximab plus chemotherapy. Br J Canc.

[CR10_242] Douillard JY (2000). Irinotecan combined with fluorouracil compared with fluorouracil alone as firstline treatment for metastatic colorectal cancer: a multicentre randomised trial. Lancet.

[CR11_242] Giacchetti S (2000). Phase III multicenter randomized trial of oxaliplatin added to chronomodulated fluorouracil- leucovorin as first-line treatment of metastatic colorectal cancer. J Clin Oncol.

[CR12_242] Giantonio BJ (2007). Bevacizumab in combination with oxaliplatin, fluorouracil, and leucovorin (FOLFOX4) for previously treated metastatic colorectal cancer: results from the eastern cooperative oncology group study E3200. J Clin Oncol.

[CR13_242] Goldberg RM (2004). A randomized controlled trial of fluorouracil plus leucovorin, irinotecan, and oxaliplatin combinations in patients with previously untreated metastatic colorectal cancer. J Clin Oncol.

[CR14_242] Hurwitz H (2004). Bevacizumab plus irinotecan, fluorouracil, and leucovorin for metastatic colorectal cancer. N Engl J Med.

[CR15_242] Khambata-Ford S (2007). Expression of epiregulin and amphiregulin and *K*-*Ras* mutation status predict disease control in metastatic colorectal cancer patients treated with cetuximab. J Clin Oncol.

[CR16_242] Lenz HJ (2006). Multicenter phase II and translational study of cetuximab in metastatic colorectal carcinoma refractory to irinotecan, oxaliplatin, and fluoropyrimidines. J Clin Oncol.

[CR17_242] Lievre A (2006). *Kras* mutation status is predictive of response to cetuximab therapy in colorectal cancer. Cancer Res.

[CR18_242] Lièvre A (2006). KRAS mutation status is predictive of response to cetuximab therapy in colorectal cancer. Cancer Res.

[CR19_242] Lievre A (2008). *Kras* mutations as an independent prognostic factor in patients with advanced colorectal cancer treated with cetuximab. J Clin Oncol.

[CR20_242] Lièvre A (2008). KRAS mutations as an independent prognostic factor in patients with advanced colorectal cancer treated with cetuximab. J Clin Oncol.

[CR21_242] McLellan EA (1993). High frequency of K-ras mutations in sporadic colorectal adenomas. Gut.

[CR22_242] Rougier P (1998). Randomised trial of irinotecan versus fluorouracil by continuous infusion after fluorouracil failure in patients with metastatic colorectal cancer. Lancet.

[CR23_242] Saltz LB (2000). Irinotecan plus fluorouracil and leucovorin for metastatic colorectal cancer. N Engl J Med.

[CR24_242] Saltz L (2001). Cetuximab (IMC-C225) plus irinotecan (CPT-11) is active in CPT-11-refractory colorectal cancer (CRC) that expresses epidermal growth factor receptor (EGFR). Proc Am Soc Clin Oncol.

[CR25_242] Saltz LB (2004). Phase II trial of cetuximab in patients with refractory colorectal cancer that expresses the epidermal growth factor receptor. J Clin Oncol.

[CR26_242] Van Cutsem E, Geboes K (2007). The multidisciplinary management of gastrointestinal cancer. The integration of cytotoxics and biologicals in the treatment of metastatic colorectal cancer. Best Pract Res Clin Gastroenterol.

[CR27_242] Van Cutsem E (2009). Cetuximab and chemotherapy as initial treatment for metastatic colorectal cancer. N Engl J Med.

